# A Narrative Review Discussing the Efficiency of Personalized Dosing Algorithm of Follitropin Delta for Ovarian Stimulation and the Reproductive and Clinical Outcomes

**DOI:** 10.3390/diagnostics13020177

**Published:** 2023-01-04

**Authors:** Bogdan Doroftei, Ovidiu-Dumitru Ilie, Nicoleta Anton, Olivia-Andreea Marcu, Ioana-Sadyie Scripcariu, Ciprian Ilea

**Affiliations:** 1 Faculty of Medicine, University of Medicine and Pharmacy “Grigore T. Popa”, University Street, no 16, 700115 Iasi, Romania; 2 Clinical Hospital of Obstetrics and Gynecology “Cuza Voda”, Cuza Voda Street, no 34, 700038 Iasi, Romania; 3 Origyn Fertility Center, Palace Street, no 3C, 700032 Iasi, Romania; 4 Department of Biology, Faculty of Biology, “Alexandru Ioan Cuza” University, Carol I Avenue, no 20A, 700505 Iasi, Romania; 5 Department of Preclinics, Faculty of Medicine and Pharmacy, University of Oradea, December 1 Market Street, no 10, 410068 Oradea, Romania

**Keywords:** infertility, follitropin delta, Rekovelle^®^, FE 999049, controlled ovarian stimulation, pregnancy outcomes

## Abstract

**Background:** Follitropin delta is the third recombinant human follicle-stimulating hormone (r-hFSH) expressed in a host cell line of human fetal retinal origin that currently emphasizes that the actual tendency of administration is a personalized dosing algorithm based on the anti-Müllerian hormone (AMH) and body mass index (BMI) for ovarian stimulation. **Methods:** In this context, we aimed, in the present manuscript, to gather all available data published between 2018–2022 regarding the co-administration and administration of follitropin delta and the clinical outcomes reported following an in vitro fertilization (IVF). **Results:** Follitropin delta is non-inferior in contrast to its previously launched agents for ovarian stimulation, enhancing a similar-to-superior response reflected by both the reproductive and pregnancy outcomes in parallel with a low risk of ovarian hyperstimulation syndrome (OHSS), being well tolerated. The body weight and AMH level are factors that may influence the outcome in a patient. Despite controversy and results that refute these arguments on several occasions, follitropin delta exceeds the benefits of conventional dosing with either follitropin alfa or follitropin beta. Thus, all post hoc, derived analyses and subsets of patients that participated in subsequent studies support this statement. **Conclusions:** Despite the relatively limited spectrum of data in the current literature, most authors brought potent proof, supporting the subsequent use of this drug depending on the patient’s profile and overcoming ethnic-related limitations. Although others contradict these observations, this topic and drug possess substantial potential, which is why additional studies are mandatory to fill the existing gaps in our knowledge and expand these experiences at a larger scale supported by the obtained reproductive and clinical outcomes that clearly indicate an overcoming of all limitations.

## 1. Introduction

Retrospectively, nearly one century has passed since the primordial observations made by Aschheim and Zondek in 1927 about the presence of a gonad-stimulating substance identified later as human chorionic gonadotropin (hCG) in the blood and urine of pregnant women [1,2]. Until the consecration of the Aschheim–Zondek reaction on a large scale as a reliable test for pregnancy [3], Seegar Jones ascertained in the 1940s that hCG is produced in the placenta [4].

Zondek proposed two years later, in 1929, founded on Smith’s research, that follicle-stimulating hormone (FSH) and luteinizing hormone (LH) are hormones synthesized at the level of the pituitary gland, both engaging the gonads [5,6,7]. From that point onwards, it was hypothesized that their biological activity might be valuable as a treatment for infertility and sparked tremendous efforts to extrapolate all knowledge into clinical practice despite the numerous limitations that occurred [8].

With the advent of recombinant DNA technologies, the field of reproductive medicine witnessed the fulminant development of novel agents, encouraging the transition from ‘’standardization’’ to ‘’individualization’’. In this manner, the development was giving birth to proteins through biological processes to obtain products with high purity in large volumes and without variability in composition that nowadays are destined to treat both male and female infertility [2].

In this context, three r-hFSHs were designed and used within current IVF protocols, with Japan becoming the first country to incorporate rFSH for controlled ovarian stimulation (COS) in assisted reproductive technologies (ART), while the fourth is under development [9].

Follitropin alfa and follitropin beta are constructed in Chinese hamster ovary cell lines and transfected, either individually to co-amplify [10] or as a single expression vector [11] for the α- and β-FSH genes, having a mean FSH activity that varies which further influences the protein content per injection [12]. Even though these two r-hFSHs exist under different commercial name(s), the safety panel in terms of side effects is minor and acceptable, among which pain at the site injection, headache, ovarian cysts, and mild-to-moderate OHSS were frequently encountered [5].

Due to characteristics in the production and purification, follitropin alfa and follitropin beta undergo a distinct glycosylation process and have separate sialic acid residue arrangements and isoelectric coefficients. More specifically, follitropin alfa is slightly more acidic, whereas follitropin beta is more basic, which is reflected in the biological activity and overall metabolic clearance [11,13,14]. This process may be the result of the isoelectric point band (pI) for follitropin alfa and (4–5 and 3.5–5.5) for follitropin beta and the consequence of the presence of fewer isoforms with a pI < 4 [13].

Interestingly, there exist post-translational changes in the biosimilar follitropin alfa products (Ovaleap^®^ and Bemfola^®^) with regards to the reference (GONAL-f^®^), apparently constituting a characteristic that is methodology-related despite the usage of the same cell line. In this context, these discrepancies stood in the higher antennarity, sialylation and batch-to-batch variability, and sialic acid N-glycolyl neuraminic in comparison with the reference [15].

The fourth r-hFSH is follitropin epsilon (Glycotope^®^), a myeloid leukemia-derived cell line with a totally different profile from follitropin alfa and follitropin beta. It has a high degree of bisecting N-acetylglucosamine, antennarity, and sialylation, especially in acidic isoforms and fucosylated, with a ratio of 2,3 to 2,6 sialylation in a 1:1 ratio that currently is not marketed [16]. Therefore, in 2016 the European Medicines Agency (EMA) approved follitropin delta (FE 999049) as an improvement compared to its predecessors, follitropin alfa and follitropin beta, and corifollitropin alfa, which dependent on the patient’s medical profile [2,9].

More precisely, follitropin delta is a novel h-rFSH expressed in a host cell line of human fetal retinal origin (PER.C6^®^, Crucell) commercialized as Rekovelle^®^ and distributed by Ferring Pharmaceuticals. Mechanically speaking, the α and β subunits in terms of the amino acid sequence are identical to those of human endogenous FSH, a mechanism mediated via the binding affinity to the FSH receptors found in the ovary that initiate intracellular cascades responsible for triggering several processes associated with the maturation of Graafian follicle and granulosa cell estrogen generation [2].

Additional in vitro experiences in the field have proven that follitropin delta is equivalent to follitropin alfa in non(human) cell lines [17] and pharmaco-kinetics (PK)—dynamic (PD) profiles being likely to contribute to the outcomes in women [18] with a risk/benefit ratio considered to be positive including headache, OHSS, pelvic discomfort and pain and, in some cases, associated with adnexa uteri, nausea, and fatigue.

The main difference between follitropin alfa and follitropin delta resides in their glycosylation nature—the latter having a superior proportion of tri- and tetra-sialylated glycans and also in 2,3- and α2,6-linked sialic acid in contrast with follitropin alfa which only has α2,3-linked sialic acid. As a result, follitropin delta possesses a unique pharmaco-PK/PD feature, suggested by the longer half-life, slow clearance, and higher response [17].

It has been discussed recently that follitropin delta cannot be substituted with follitropin alfa in clinical practice because the latter is calculated with the Steelman–Pohley bioassay, uses an international reference standard, and cannot be dosed according to its or specific bioactivity, as other follitropins, but instead is dosed by mass (µg) [18].

Concomitantly with the administration according to a personalized dosing algorithm depending on the patient’s AMH as a predictor for the ovarian reserve and response to the exogenous supply and BMI after the gradual administration of follitropin delta, this manuscript aims to gather all data published in the last half a decade concerning the clinical outcomes surrounding follitropin delta on women undergoing IVF.

## 2. Methodology

### 2.1. Rationale of the Study

The concept behind selecting studies published in the last half a decade stands in the fact that Rekovelle^®^ received marketing authorization in 2016, and this is why we decided to not only retrieve and offer an updated support pillar for both clinicians and researchers but also highlight the gaps existing in our knowledge and the necessity of further studies to expand this direction of activity.

### 2.2. Database Searching Strategy

Data until inception (October 2022) for conducting this manuscript were selected from four databases: particularly, PubMed/Medline, ISI Web of Knowledge, Scopus, and ScienceDirect.

The searching strategy was based on several keyword combinations implied to cover as much information as possible such as “humans”, “infertility”, and “ovarian stimulation” in parallel with “follitropin delta”, “Rekovelle^®^”, and “FE 999049”.

The adopted PubMed/Medline string was as follows: humans[Title/Abstract] OR infertility[Title/Abstract] OR ovarian stimulation[Title/Abstract] AND follitropin delta[Title/Abstract] AND Rekovelle^®^ [Title/Abstract] AND FE 999049[Title/Abstract].

Independently of PubMed/Medline, where we limit from the beginning searches to strictly studies conducted on human patients and English as the primary language, in ISI Web of Knowledge, Scopus, and ScienceDirect, we restricted the returned entries to research articles.

### 2.3. Inclusion Criteria

All potentially eligible studies must be written in the English language, are not to be published before 2018, and report experiences on human patients.

### 2.4. Exclusion Criteria

Articles written in another language than English, case series/reports, standard reviews or systematic reviews, meta-analyses, letters to the Editor, editorials, comments, opinions, conference posters/abstracts, work protocols, computational simulations, and preprints were banned.

### 2.5. Study Selection

All five authors reviewed the information in the titles and abstracts of the retrieved results. Those considered eligible were screened based on the complete content. We solved discrepancies by common consent between B.D., O.-A.M., and C.I.

### 2.6. Number of Entries

**Figure 1:**
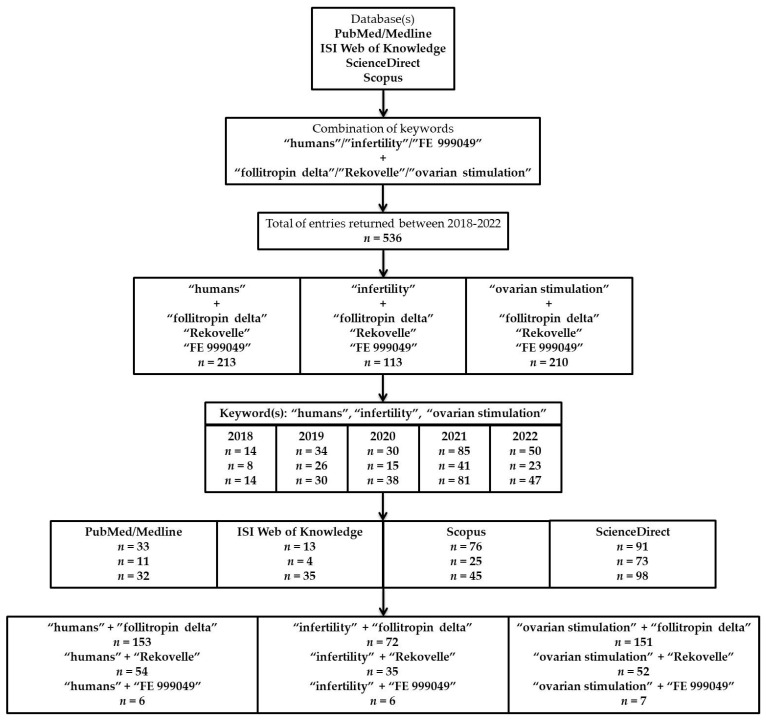
A flowchart of the study design, strategy, studies, and results that were included in the manuscript.

We identified a total of n = 536 entries during the pre-established interval (2018–2022), from which n = 213 when using the combination of “humans” + “follitropin delta”, “Rekovelle^®^”, and “FE 999049”; n = 113 for “infertility” + “follitropin delta”, “Rekovelle^®^’, and “FE 999049”; and n = 210 when applying “ovarian stimulation” + “follitropin delta”, “Rekovelle^®^”, and “FE 999049”. An overview of all studies and entries based on the combination of keywords and databases searched is presented in Figure 1.

Following the centralization of all potentially eligible studies and removing duplicates according to the exclusion criteria implied, a total of n = 21 studies met the initial criteria for further assessment. Based on the second evaluation, only n = 8 were further considered since the authors investigated several parameters regarding the clinical outcomes. From the remaining n = 13 studies, n = 4 were excluded due to design incompatibilities, while n = 9 were used only as informatic support throughout this manuscript to either bring an alternative perspective or confirm/refute the results presented by other teams.

A stratification of the studies based on the number of patients, allocation, reproductive outcomes, dose concentration, clinical outcomes (vital pregnancy, clinical pregnancy, ongoing pregnancy, multiple pregnancies, live birth, and live birth after 4 weeks), and year of publication are presented in Table 1.

From the n = 8 studies summarized in Table 1, n = 1 were published in 2018 [19], n = 2 in 2020 [20,21], n = 3 in 2021 [22,23,24], and n = 2 in 2022 [25,26], with a total of n = 3750 women being included in these studies. Despite the exceptionally scarce data in the current literature, several clinical trials are currently underway which were found in distinct stages. An overview of all clinical trials found in the recruiting phase is offered below in Table 2.

The main inclusion criteria established for patients to be eligible are tubal infertility, unexplained infertility, endometriosis (EMS) stage I/II, or partners diagnosed with male factor infertility, whereas the main exclusion criteria revolved around (EMS) stage III/IV, three or more COS, history of recurrent miscarriage, and use of hormonal preparations excepting thyroid medication.

## 3. Results

The two major and most prominent research projects that constitute the core and support pillars of the actual field on the use of follitropin delta within ART as an integrative companion in IVF protocols are The Evidence-based Stimulation Trial with Human rFSH in Europe and Rest of (ESTHER-1) (NCT01956110) and ESTHER-2 (NCT01956123). Although a handful of studies were published [31,32,33,34,35], crucial subsidiary data complete our knowledge and expand the current applicability sphere, the evidence being discussed below for a comprehensive view [19,22,25,36,37].

ESTHER-1 (NCT01956110) trial was the most recognizable program that reunited n = 1329 participants from twelve countries and thirteen specialized centers from Europe and North and South America, conducted between 2013–2017, whose objective was to compare the efficiency and safety of follitropin delta compared with conventional dosing of follitropin alfa in women undergoing IVF. Based on the design established by the authors and the interest in the ongoing implantation and pregnancy rates following randomization, they divided the eligible women into two arms: n = 665 follitropin delta and n = 661 follitropin alfa. In this context, a maximum of 12 µg follitropin delta and 450 IU from a standard of 150 IU led to higher rates in both categories (30.7% vs. 31.6%; 35.2% vs. 35.8%) but were similar in terms of live births (29.8% vs. 30.7%). This approach enhanced a response per oocytes (8–14 oocytes) (43.3% vs. 38.4%), fewer poor/excessive responses (<4 oocytes when AMH < 15 pmol/L—11.8% vs. 17.9%)/(≥15 or ≥20 oocytes when AMH ≥ 15 pmol/L—27.9% vs. 35.1%, 10.1% vs. 15.6%), and fewer actions carried out to prevent OHSS (2.3% vs. 4.5%), despite oocyte yielding, blastocysts number, and gonadotropin use (10.0% vs. 10.4%, 3.3% vs. 3.5%, 90.0% vs. 103.7%) [34].

However, additional studies did not include Asia because several teams from the USA and UK uncovered ethnic limitations in the treatment’s efficiency due to patient-related factors and depending on the diagnostic markers of the ovarian reserve as well [38,39,40], although the idea to perform investigations across distinct nationalities from specific geographic locations is supported.

Subsequently, it derived ESTHER-2 (NCT01956123) which included n = 513 women, crystallized under the publication of numerous articles. Shortly after the emergence of the first set of data offered by Nyboe Andersen et al. [34], Bosch and his colleagues [19] extracted a subset of n = 701 ESTHER-1 participants to evaluate the immunogenicity of follitropin delta in repeated ovarian stimulation cycles and incorporated it within the study. They argue that follitropin delta is well tolerated and has low immunogenicity potential, even in those with primary anti-FSH antibodies, and, thus, did not require an elevated immune response. Their results are congruent with previous evidence revealing a reactivity in cycle 1 and cycle 3 of 1.1% and a slight difference from women that underwent cycle 2 with only 0.8%. Nevertheless, the reproductive outcomes highlight similar results, particularly mean oocyte retrieval in cycle 2 and cycle 3 of 9.2–8.6 and 8.3–8.9.

As mentioned and expected, ESTHER-1 and ESTHER-2 sparked a constantly growing interest in the field based on the results of Nelson et al. [33]. In contrast with the parameters in other manuscripts, these authors follow the impact of a dosing algorithm that might indicate the inter- and intracycle variability through multiple measurements of AMH in n = 1326 women. Although the correlation coefficient (r) was 0.92, showing a strong correlation within the individual after repeated serum AMH measurements, the AMH had a confined impact within subject variation (coefficient of variation (CV)) that was 23%, a low time-dependent decline (mean of 6% per year) without systematic variation across menstrual cycles. Regardless of the AMH screening value in or at the initiation of protocol, the number of oocytes does not vary beyond 1 in 95% of all women, <15 pmol/L (93% with an attaining number of oocytes in 97% of cases) or ≥15 pmol/L (80% receiving ±1.5 μg that would attain ±1 oocyte in 90% of cases).

Besides the studies of Bosch, Nelson et al. [19,33], another concern arose since the establishment of the large-scale applicability of follitropin delta. This time it had as an objective the efficiency in preventing the risk of developing OHSS in a cumulative manner, concerning a second analysis of results in n = 1326 according to Nelson et al. [33] and n = 701 according to Bosch et al. [19], in women subjected to sequential ovarian stimulation. In this way, Fernández-Sánchez et al. [37] showed that follitropin delta significantly lowers moderate/severe OHSS. This discovery is reflected in the preventive intentions, (*p* = 0.018) by comparison with conventional dosing following up to three ovarian stimulation cycles. It is worth mentioning that patients with high AMH (*p* = 0.012) had the most meaningful benefit but with the mention that separate evaluation of follitropin delta still notably reduced the incidence of moderate/severe OHSS (*p* = 0.036) and preventive intentions (*p* = 0.044) compared with follitropin alfa.

However, when evaluating the performance of commercial AMH assays, it is recommended that it be performed using different assays because a variation from 28% to 163% was pinpointed by Bungum et al. [41] and replaced with more sensitive AMH assays for intra- and inter-cyclic variations.

Continuing with this concept, Višnová et al. [22] intended to explore how AMH of so-called high responders might impact the chance of pregnancy. Patients treated with individualized follitropin delta had, in the end, follicles measuring ≥ 12 mm (12.1 vs. 18.3), higher progesterone (P4) levels (>3.18 nmol/L) (27.3% vs. 66.7%), and oocyte retrieved (9.3 vs. 17.9) (*p* < 0.001). As emphasized in other studies, the risk for developing OHSS in conventional follitropin alfa was up to three times higher (16.0% vs. 5.1%) (*p* = 0.025), as well as early moderate/severe OHSS (26.7% vs. 7.7%) (*p* = 0.001).

Fortunately, another team in the name of STORK group tried to overcome the limitations regarding the nationalities profile, and they started a series of experiments with the aim of testing, once again, the efficacy and safety of using follitropin delta for ovarian stimulation through an individualized regimen with fixed doses in Japanese women. Analogous to the results of Fernández-Sánchez et al. [37], in this case, Ishihara et al. [23] (NCT03228680) successfully offered additional data demonstrating a non-inferior potential of follitropin delta for the number of retrieved oocytes compared with follitropin beta (9.3 vs. 10.5%). In other words, this drug modulates the occurrence of OHSS by almost half, with the incidence reaching 11.2% vs. 19.8% (*p* = 0.021), particularly for OHSS of any grade—respectively, 7.1% vs. 14.1% (*p* = 0.027) for moderate/severe OHSS.

It is worth noting that Arce et al. [31] conducted a post hoc analysis of two previous RCTs ((NCT01426386) (NCT01956110)) to investigate whether follitropin delta promotes a comparable response to 150 IU/day follitropin alfa. They observed that, by analyzing the ovarian response via IVF/intracytoplasmic sperm injection (ICSI) in n = 1591 women and estimating a dose in the range of 9.5–10.4 µg to 150 IU, it yielded the same number of oocytes. Such findings were extrapolated in patients with a normal or high ovarian reserve that underwent 9.7 µg and 9.3 µg, respectively, compared with 10.0–10.3 µg in phases II and III.

Another post hoc analysis was conducted by Ishihara et al. [35], also from two former RCTs ((NCT03228680), (NCT01956110)) reuniting n = 800 IVF/ICSI patients—n = 170 Japanese women and n = 630 White women—to assess the differences of ovarian response. Body weight is a definitory variable in Japanese women because it affected the total follitropin delta dose as opposed to conventional dosing (83.5% vs. 90.2%). Moreover, the serum FSH concentration at the end of stimulation did not differ significantly between the groups (14.3 vs. 14.0 IU/L), but the serum estradiol (E2) concentration was higher in Japanese women (6517 vs. 5298 pmol/L) (*p* < 0.0001). The number of retrieved oocytes was similar (9.3% vs. 9.5%), also among all potential low and high responders (7.2% vs. 7.6%—10.8% vs. 11.0%). There was a statistically significant difference in the serum E2 concentrations at the level of ovarian response in Japanese women (*p* = 0.024) but also for the risk of early OHSS (10.0% vs. 2.2%) (*p* = 0.0124) at similar serum E2 concentrations (*p* = 0.0137).

The fruition of ESTHER-1 and ESTHER-2, defined by Havelock et al. [32], describes both pregnancy and neonatal outcomes using fresh and vitrified/warmed blastocysts acquired through ovarian stimulation. From a total of 2719 (n = 1012 and n = 341 fresh/frozen cycles for follitropin delta, and n = 1015 and n = 351 fresh/frozen cycles for follitropin alfa) cycles performed in n = 1326 IVF/ICSI patients from a phase III trial [31,33], the cumulative rate of take-home babies was 60.3% vs. 60.7%, with a relative contribution of 72.8% from fresh cycles and 27.2% from frozen cycles. The overall rate of ongoing implantation was 32.1% across the fresh cycles in both follitropin alfa and follitropin delta, whereas it was 27.6% and 27.8% for frozen cycles with an incidence of major congenital anomalies among neonates until 4 weeks of 1.6% for follitropin delta and 1.8% for follitropin alfa.

Even though ESTHER was encouraging, it is still unclear how follitropin delta would be adopted and implemented in broader clinical practice. There is a new publication from Bissonnette et al. [36] (NCT03483545) and lately Bachmann et al. [25] discussing this matter by referring to the results obtained in ESTHER.

First, their investigation relies on the benefit of a mixed protocol and a comparison of n = 110 participants subjected to the co-administration of follitropin delta with highly purified human menopausal gonadotropin (HP-hMG) to a historical control group from ESTHER-1. The Menopur and Rekovelle Combined Study (MARCS) revolves around a specific strategy, among which 75 IU and <12 µg were the starting doses, with the possibility of adjusting based on the weight, <100 kg for 150 IU and 225 IU at ≥100 kg. Therefore, there was a statistically significant higher mean when stratified by age in the number of retrieved oocytes and of high-quality blastocysts compared with ESTHER-1. More specifically, the rates of patients triggered with a gonadotropin-releasing hormone agonist in MARCS cohort was 43% vs. 2.3% in ESTHER-1, while the incidence of OHSS was 9.3% in contrast with 2.6%, without any case of moderate/severe OHSS and with 1.4% in ESTHER-1 [25].

Our group demonstrated [42] that women under 35 years (n = 122) old have higher chances of achieving pregnancy compared with their older counterparts over 35 years old (n = 83), as suggested by the follicles > 18 mm yielded, retrieved oocytes, and D3 embryos—1719 vs. 814, 1279 vs. 612, and 677 vs. 301. We noted n = 45 pregnancies in women < 35 years old, while in elderly counterparts, only n = 13, with n = 6 ongoing pregnancies in the younger group. Unfortunately, we had to stop n = 9 pregnancies in women < 35 years old, from which n = 8 were unsuccessful, including n = 1 awaiting and n = 1 abortion, respectively. On the contrary, we obtained a multiple pregnancy in a 39-year-old lady and half of the rate of unsuccessful pregnancies in n = 4. Similar to the figures reported in the younger women, we had n = 1 awaiting response and n = 2 abortion.

Finally, an assessment of the follitropin delta and the confirmation in routine clinical practice tested recently show a mean number of oocytes of 11.2%, from which 42.1% of patients had between eight to fourteen oocytes. As opposed to the study of Bissonnette et al. [36], Bachmann et al. [25] demonstrate that their findings are in line with ESTHER-1, despite the variations of AMH from 3.6 pmol/L (2.5%) to 40 pmol/L (19.7%), from which 79.7% had an AMH above 14.5 pmol/L.

Congruent with previous aspects extensively treated in the above chapter, we found it suitable to present additional studies to mark follitropin delta that might contribute to achieving a healthy pregnancy in couples seeking specialty care.

As expected, the increase in daily doses of follitropin delta ensures a higher number of oocytes retrieved (7.0, 9.1, and 11.6) among all tested groups compared with recipients of follitropin beta as a reference, also indicated by the serum E2 concentration (5521.0 pmol/L, 6178.1 pmol/L, and 7573.0 pmol/L) and relatively low rate of OHSS—8% for 6 µg/day and 9 µg/day and 13% for 12 µg/day. This study accomplished with Japanese women by Ishihara et al. [20] (NCT02309671) strengthens the findings consistent with those obtained from prior trials in Europe and are in accordance with the data discussed above.

Subsequently, Haakman et al. [21] demonstrate that, besides the similar rates in clinical pregnancies, there was no statistically significant difference in embryo parameters in D3 (0.50 vs. 0.54, 0.25 vs. 0.20 median for good and intermediate quality embryos). Even though the first analysis noted that there was a low proportion of good quality blastocysts in women receiving follitropin delta compared with the control (0.11 vs. 0.22), this was no longer applicable on D3 after fertilization vitrification and the transfer cycles exclusion (0.26 vs. 0.33).

Interestingly, Qiao et al. [24] (NCT03296527) contradict the existing data by demonstrating a statistical significance (*p* < 0.001) on the retrieved oocytes attributed to follitropin delta in contrast with conventional follitropin alfa (10.0 vs. 12.4) in Asian women, with those treated with follitropin delta yielding on average two more oocytes (9.6 vs. 7.6%) in low responders when AMH < 15 pmol/L and three fewer oocytes on average (10.1 vs. 13.8) in potential high responders when AMH ≥ 15 pmol/L, but the excessive response occurred less frequently in follitropin delta-treated patients than in those receiving follitropin alfa (≥15 oocytes: 20.2% vs. 39.1%; ≥20 oocytes: 6.7% vs. 18.5%—*p* < 0.001). Moreover, the authors stated that they succeeded in reducing the incidence of OHSS and/or of preventive measures (9.6% vs. 5.0%—*p* < 0.001) due to the use of follitropin alfa but also reduced the number of total gonadotropin (109.9 vs. 77.5—*p* < 0.001).

In addition to the attempt of Bachmann et al. [25] to combine follitropin delta with HP-hMG, Fernández-Sánchez et al. [26] (2017-003810-13 (EudraCT Number)) postulate how CG beta alongside follitropin delta might increase the number of good-quality blastocysts in a long gonadotropin-releasing hormone (GnRH) protocol. Although the number and size of follicles were similar on D6 among the treated groups, a dose-related reduction of intermediate follicles was noted (12–17 mm) compared to the placebo group. On the other hand, the number of follicles ≥ 17 mm was appropriate between the placebo and CG beta groups, with a decreased number of intermediate follicles (12–17 mm) and fewer oocytes (9.7–11.2) in all CG beta compared with the follitropin delta (12.5). Taken in a cumulative manner, the mean number of good-quality blastocysts ranged from 2.1 to 3.0 in CG beta, whereas in the follitropin delta group, there was 3.3. The incidence of OHSS remained low, varying from 2.0% to 10.3% in CG beta, to 11.5% in follitropin delta.

Longobardi et al. [43] reported a study, in which they recruited n = 60 fertility nurses and n = 120 women with infertility, showing that preparation of the pen injector may reduce the risk of handling errors, related to a reduction in treatment-related anxiety. They reveal that the GONAL-f^®^ pen is preferred in both naive and experienced women over other injection devices such as Bemfola^®^, Ovaleap^®^, and Rekovelle^®^.

Regarding the concerns of the ongoing clinical trials, it appears, based on their study design, that it will assess the usage patterns, efficacy, and safety of Rekovelle^®^ in women undergoing COS [29], up to two cycles of IVF/ICSI [28], by measuring the number of eggs constructed at the beginning of the procedure following a dose of 15 µg Rekovelle^®^ by comparison to 225 IU GONAL-f^®^ [27] and the non-inferiority potential of Rekovelle^®^ in contrast with GONAL-f^®^ [30] with respect to the ongoing pregnancy rate.

### Strengths and Limitations of the Study

This manuscript offers an updated perspective on the settings, protocols and methodologies applied regarding follitropin delta usage in clinical practice by different teams and presents both the reproductive and clinical outcomes. Although this topic objectively covers and treats comparatively the existing evidence from the current literature, we did not perform a quantitative meta-analysis by design due to the high heterogeneity of the data and the relatively small number of studies, rather applying a much more conventional approach. This decision may be derived from the fact that considered eligible studies were either post hoc analyses or had a new parameter to assess to primarily ensure reproducibility.

## 4. Discussion

Former analyses that aimed to clarify the immunogenicity of both follitropin alfa and follitropin beta demonstrated the inexistence of anti-FSH antibody production [44,45] with results that correspond with others concerning neutralizing capacity for neither daily administration nor long-acting rFSH formulations [46,47,48]. Tangent with the preceding efficacy trial [34], in which there was carried out an assessment of anti-FSH at baseline before the first cycle subsequently certifies the occurrence of natural anti-FSH antibodies [49,50,51]. Considering that a reduction of the pregnancy rate in the repeated cycles is not uncommon, as it has been already described [47,48,52] and sustained [46], this phenomenon can be explained by the participants’ ability to choose the cryopreservation instead of a new stimulation cycle for women with good-quality blastocysts. While the ovarian response was similar regardless of the dose increment [53,54], it promoted satisfactory outcomes with a low risk of OHSS.

Interestingly, after conducting two studies on Japanese women, Ishihara et al. [20,23] reveal antithetical findings that are somewhat not surprising, most notably the ovarian response, which is tangent with two previous investigations [55,56], further indicating a personalized pattern observed in non-Japanese patients categorized as poor responders as they usually yielded with one more oocyte than the Asian population [34] and with above-average pregnancy rates reaching up to 22% according to registry data in fresh cycles with SET [57]. One aspect that has been put forward is the associated risk of OHSS which favors more toward the use of follitropin delta since the risk appears to be correlated with the oocyte retrieval (≥15 oocytes) between 20% to 22% in Japanese patients [58,59,60] and is much lower than in European countries [55]. Among the developmental risk factors stands polycystic ovary syndrome (PCOS), low BMI, high or rapidly rising E2 after stimulation, and a high number of oocytes retrieved [61]. Besides the low risk of immunogenicity [17,19,34,55,62] and the very different pattern of oocyte yielding between low and high ovarian reserve patients treated with the same dose, it should be considered that increasing the gonadotropin dose may not result in overcoming the risk of poor response or achieve a pregnancy and live birth [63]. One utmost peculiarity is the psychosocial attribute that should not be omitted since it proved to be crucial in knowing patients’ preferences and experiences, including the reasons for the decision of not further pursuing care or cycles to fulfill the needs [64,65]. Despite this evidence, there are several limitations of these studies conducted by Ishihara [20,23] that would allow a prudent comparison of treatment results such as pregnancy and OHSS rates between follitropin beta and follitropin delta, such as the lack of investigation of frozen cycles to cumulative live birth.

One additional manuscript conducted by Qiao et al. [24] validated the results of Ishihara and Arce [23] in Asian women for the usage of individualized gonadotropin dosing, extrapolating this approach one step further with respect to parameters of interest, including the ongoing pregnancy rate, even exceeding beyond the recognized advantages from previous studies [19,23,34]. As concluded in other trials, the higher oocyte yield does not necessarily reflect a better outcome in fresh cycles; increased gonadotropin consumption in a conventional strategy ensures a higher occurrence of P4 but appears to be linked with a reduced chance of pregnancy [66]. Nonetheless, as exposed throughout this extensive debate, the risk of OHSS was lower when using individualized follitropin delta with approximately 25% to 50% compared with follitropin alfa, as described in other RCTs [23,34].

Although the profile of follitropin delta corresponded with the other two variants, Haakman et al. [21] documented a relative comparison in clinical pregnancies indices with fresh transfer, with ESTHER-1 demonstrating an enhanced safety profile of follitropin delta, to identify the number of excessive stimulation responses and measures to prevent OHSS [19,34,37]. To strengthen the position and support pillar of this fundamental study, the results of other parameters of interest are discussed, such as clinical implantation and pregnancy between the analyzed groups, among which ongoing implantation, pregnancy, and live birth rates were in line with ESTHER-1 [34]. Nevertheless, oocyte yielding was equivalent under comparison, but Arce et al. [55] refuted this data, showing a positive relationship between follitropin delta dose and oocyte number without translating an increase in the blastocysts number. Analogous to the abovementioned studies, this one also has limitations that revolve around the differences in demographic characteristics, and the patients’ ovarian reserve was based on antral follicle counts (AFCs) since AMH serum levels have not been available from the start [67,68].

Another set of data derived from the study of Bachmann et al. [25] indicates the accession of the preferred number (8–14) of oocytes at an AMH median of 25.6 pmol/L in the first cycles (42.1%), which is higher than in ESTHER-1, but matches with that from the ESTHER-1 (43.3%) [34], with a negligibly extended number of days and circumstances where patients with low and/or very low AMH concentrations had a small number of oocytes by comparison with those with high concentrations [69,70,71,72]. The subsequent stratification by age regarding the pregnancy rate has proven to be higher than figures reported in the Deutschen IVF-Register (D.I.R) database four years ago in Germany for the same age interval (44.4% vs. 38.5% for 30–34 years old and 37.9% vs. 32.2% for 35–39 years old) [73]. In the same report, it is stated that an average of 1.8 embryo(s) were transferred via IVF/ICSI in 2017 compared with 1.4 embryo(s), according to Bachmann et al. [25], and a higher pregnancy rate per transfer than D.I.R, with a declarative 61.2% SET as opposed to 95.9% in ESTHER-1. Numerous legislative-related limitations were also identified in this experiment, especially concerning the blastocyst culture, embryo selection, and requirements for cryopreservation at the two pronuclear (2PN) stages. Fernández-Sánchez et al. [26] pursue minimizing the endogenous LH by adopting a strategy involving the concomitant administration of follitropin delta with hCG (CG beta), arguing that a proportional increment of serum CG beta concentrations with CG beta dose reaches a steady trend on stimulation D6, with observations already documented in female volunteers [74]. One of the most important aspects covered in this study is the broad range of CG beta doses used to enable the optimal dose selection, but a diminish in the number of intermediate follicles was observed, data which is in antithesis with a small clinical trial in which urinary hCG divided into three separate doses were added into a daily dose of 150 IU rFSH [75]. It is vital to note that such a topic started to gain attention since 1994 when the first proofs were brought into the light on murine models when tested the effect of rFSH and urinary hCG on follicular growth and atresia in immature hypophysectomized rats [76]. Another research study that shows an influence upon antral follicles in an FSH dose-dependent manner stands on the experimental models in vivo highlighting that optimal follicular evolution requires a mixture of 20 IU FSH and 1 to 10 IU hCG but not higher than 50 to 100 IU because it causes a decline in the number of embryos [77]. Dose adjustments are mandatory given that serum P4, after the last follicular growth with 250 µg r-hCG, displayed a dose-dependent lowering on the oocyte retrieval day. This event appears to be correlated with intermediate follicles’ reduction, and, furthermore, for P4, production per follicle declined in a CG-dose-dependent manner, with causality related to too high and/or long exposure to CG beta via a down-regulation of the LH receptor [78,79,80,81]. The process of the LH or hCG ability to inhibit multiple follicular development has been treated on multiple occasions over the years by several authors dating back to more than one decade, among which some of them refer to the World Health Organization (WHO) type I and II anovulation due to exposure to relatively high doses of recombinant LH during the late follicular phase [82,83]. For example, Filicori et al. [84,85,86] deepen this matter by estimating the impact LH had on the activity by using hMG or hCG in typical ovulatory women that follow intrauterine insemination (IUI) or ovarian stimulation for ART, noting a continued reduction in the small follicles (<10 mm) number due to androgen-mediated follicle atresia. In another circumstance, the increment of hCG from 100 to 400 IU led to a low number of follicles that measure between 10–14 mm, with the mention that 400 IU hCG had an influence on the oocyte and embryo yielding (EP2292252B1)—knowing, from a recent study, that FSH and LH receptors’ expression is related to the follicles’ diameters in human granulosa cells [87]. While the elevated levels of androgens cause atresia [88] during ovarian stimulation, this is not applicable for FSH and estrogen which proved to be essential for follicles to escape atresia in order to reach the pre-ovulatory follicle stage [89]. However, this equilibrium may be less delicate in two case scenarios when pre-ovulatory follicles produce massive quantities of E2 in order to contain atresia, or when there are small amounts and subsidiary, androgen-dominant follicles, this hormone may take part in follicle loss [90]. Cumulatively, Fernández-Sánchez et al. [26] set as the primary endpoint the good-quality blastocysts as others report [75] by noting a reduction of the ongoing pregnancy rate per started cycle. Irrespective of the limited experiences, the authors implied a set of inclusion criteria that restricted the participation of women with a high AMH to prevent the risk of cycle cancelation, coupling this argument with the historical comparative data since a higher ovarian response and number of oocytes following GnRH antagonist with GnRH agonist was anticipated [91,92]. Overall, the clinical outcomes were excellent in both tested groups, whereas the follitropin delta promoted an average of 12.5 oocytes—3.3 good-quality blastocysts on D5 after retrieval and a 42.9% ongoing pregnancy rate per started cycle as compared to Nyboe Andersen et al. [34]. The final remark made by the authors concerns the fact that no cycle was cancelled as a consequence of an excessive ovarian response in the placebo arm despite the tendency of OHSS occurrence as previously reported [34,37].

The final study concentrated on clarifying the issues associated with potentially high responders belonging to Višnová et al. [22] where a decrease in the mean oocytes number per started cycle was achieved from 17.9 to 9.3 between treatment schemes, the latter being similar to 9.6 documented in the ESTHER-1 [34]. Once again, these remarks tend to the administration of follitropin delta to lessen the odds of OHSS in this category if we refer to past examinations (7.7% vs. 26.7% compared with 4.4% vs. 6.7%) [34,37]. The authors additionally assert that the incidence of premature elevation in serum P4 at the end of stimulation was at least two times higher in the follitropin alfa group compared with the follitropin delta which is higher than in the literature, perhaps due to the selection of women with high AMH, but their analysis suggests little detrimental impact of these premature P4 peaks on the pregnancy rates [93,94]. The so-called high responders (49 pmol/L) were a couple of years younger than the normal individuals (16 pmol/L) and had an AMH level up to three times higher, partially defining the variability in ovarian response in which, interestingly, the analyzed patients underwent an equivalent period of nine days of stimulation as in Nyboe Andersen et al. [34], implying adjustments of daily rFSH doses to ensure the same triggering criteria that reach up to 34.5 µg translated into 500 IU per treatment cycle [31]. Although participants in this trial received a personalized dose of follitropin delta by taking into account their AMH (>35 pmol/L) and body weight (32 kg/m^2^), evidence in the field describes that women suffering from PCOS and polycystic ovaries represented a risk group for the overweight and/or obese [95]; the selection of an optimal gonadotropin dose is, therefore, a real challenge due to a negative correlation between the serum FSH level and body weight [96], low variability of serum FSH, and poor ovarian response [96,97]. Based on the actual PCOS and/or polycystic ovaries diagnostic guidelines [98] and given that women with polycystic ovaries also may have high AMH levels [99], the diagnostic AMH cut-off value(s) may depend on the age and phenotype as a worsening variable that complicates the detection of polycystic ovaries [100]. The main limitation of this research was the establishment of an AMH cut-off of 35 pmol/L, as adopted by Dewailly et al. [101], but the assessment consists of different analyzers, of which the manual AMH Gen II may have a higher sensitivity than the automated Elecsys^®^ AMH [102], with reports suggesting a cut-off value oscillating between 20 pmol/L or 25 pmol/L to categorize women with polycystic ovaries within PCOS population [103,104], valid for OHSS (≥25.35 pmol/L) as well [37].

## 5. Conclusions

Based on the aspects presented throughout this paper, follitropin delta is a safe, efficacious, and well-tolerated r-hFSH drug with low immunogenicity, even for high responders. Moreover, it may surpass the conventional dosing of follitropin alfa/beta despite some controversy that revealed the opposite and encouraged co-administration with HP-hMG or hCG beta. Overall, follitropin delta may be viewed as a successor of the other two r-hFSHs, as reflected by both reproductive and clinical outcomes and suggested by the success rates, but surely we need further testing until it can be successfully incorporated within routine clinical practice. Although, in several occasions, several teams of authors aimed to achieve this, further data, observations, and findings are mandatory, due to the limited number of studies. According to the author’s best knowledge, this is the first manuscript to objectively describe the results into this matter.

## Figures and Tables

**Table 1:** Overview of the reproductive and clinical outcomes.

Number of Patients	Allocation	Reproductive Outcomes	Dose Concentration	Clinical Outcomes	Reference
n = 701	Cycle 2 n = 252; follitropin delta n = 261; follitropin alfa Cycle 3 n = 95; follitropin delta n = 93; follitropin alfa	Cycle 2 10.2 ± 5.2 vs. 9.9 ± 4.9; follicles ≥ 12 mm 9.2 ± 4.8 vs. 8.6 ± 4.3; oocytes retrieved 56.8 ± 23.5 vs. 52.6 ± 24.3; fertilization rate 5.1 ± 3.3 vs. 4.3 ± 2.8; total embryos D3 3.9 ± 3.1 vs. 3.3 ± 2.4; good quality embryos D3 2.8 ± 2.4 vs. 2.4 ± 2.1; total blastocysts D5 1.4 ± 1.7 vs. 1.2 ± 1.6; good quality blastocysts D5 Cycle 3 8.9 ± 4.5 vs. 9.8 ± 4.8; follicles ≥ 12 mm 8.3 ± 4.0 vs. 8.9 ± 4.2; oocytes retrieved 56.3 ± 20.6 vs. 49.7 ± 24.9; fertilization rate 4.4 ± 2.4 vs. 4.4 ± 3.3; total embryos D3 3.2 ± 2.2 vs. 3.3 ± 3.0; good quality embryos D3 2.2 ± 1.8 vs. 2.4 ± 2.3; total blastocysts D5 1.2 ± 1.5 vs. 1.2 ± 1.8; good quality blastocysts D5	Cycle 2 18 µg—max dose 225 IU—max dose; 75 IU adjustments, up to max 450 IU Cycle 3 24 µg—max dose 300 IU—max dose; 75 IU adjustments, up to max 450 IU	Cycle 2 n = 82 (32.5%) vs. n = 79 (30.3%)—clinical pregnancy rate n = 74 (29.4%) vs. n = 71 (27.2%)—vital pregnancy rate n = 70 (27.8%) vs. n = 67 (25.7%)—ongoing pregnancy rate n = 69 (27.4%) vs. n = 66 (25.3%)—live birth rate n = 69 (27.4%) vs. n = 66 (25.3%)—live birth rate at 4 weeks n = 5 (7.1%) vs. n = 2 (3.0%)—multiple pregnancy rate Cycle 3 n = 31 (32.6%) vs. n = 30 (32.3%)—clinical pregnancy rate n = 26 (27.4%) vs. n = 27 (29.0%)—vital pregnancy rate n = 26 (27.4%) vs. n = 26 (28.0%)—ongoing pregnancy rate n = 25 (26.3%) vs. n = 25 (26.9%)—live birth rate n = 25 (26.3%) vs. n = 25 (26.9%)—live birth rate at 4 weeks n = 8 (30.8%) vs. n = 10 (38.5%)—multiple pregnancy rate	[19]
n = 158	n = 37; follitropin delta n = 40; follitropin delta n = 40; follitropin delta n = 41; follitropin beta	7.0 ± 4.1 vs. 9.1 ± 5.6 vs. 11.6 ± 5.6 vs. 11.0 ± 4.7; oocytes retrieved in all patients 5.3 ± 3.7 vs. 5.6 ± 3.5 vs. 9.5 ± 3.0 vs. 9.3 ± 4.3; oocytes retrieved in low AMH stratum 7.9 ± 4.1 vs. 11.2 ± 5.6 vs. 12.9 ± 6.4 vs. 11.8 ± 4.7; oocytes retrieved in high AMH stratum 3.2 ± 2.2 vs. 4.4 ± 3.1 vs. 5.9 ± 3.6 vs. 5.7 ± 3.8; fertilized oocytes in all patients 2.5 ± 2.2 vs. 2.9 ± 2.3 vs. 3.9 ± 2.3 vs. 5.4 ± 3.2; fertilized oocytes in low AMH stratum 3.5 ± 2.2 vs. 5.3 ± 3.2 vs. 7.0 ± 3.7 vs. 5.9 ± 4.1; fertilized oocytes in high AMH stratum 21.4 ± 8.5 vs. 23.6 ± 9.5 vs. 28.5 ± 10.2 vs. 27.9 ± 10.0; follicular volume in all patients 19.9 ± 8.4 vs. 18.9 ± 6.7 vs. 23.2 ± 6.3 vs. 24.7 ± 9.1; follicular volume in low AMH stratum 22.1 ± 8.6 vs. 26.4 ± 10.0 vs. 31.7 ± 10.7 vs. 29.6 ± 10.2; follicular volume in high AMH stratum	6 µg 9 µg 12 µg 150 IU	n = 9 (24%) vs. n = 8 (20%) vs. n = 13 (33%) vs. n = 8 (20%)—clinical pregnancy rate per started cycle n = 9 (35%) vs. n = 8 (26%) vs. n = 13 (41%) vs. n = 8 (26%)—clinical pregnancy rate per cycle with transfer n = 7 (19%) vs. n = 8 (20%) vs. n = 10 (25%) vs. n = 6 (15%)—vital pregnancy rate per started cycle n = 7 (27%) vs. n = 8 (26%) vs. n = 10 (31%) vs. n = 6 (19%)—vital pregnancy rate per cycle with transfer n = 6 (16%) vs. n = 7 (18%) vs. n = 10 (25%) vs. n = 6 (15%)—ongoing pregnancy rate per started cycle n = 6 (23%) vs. n = 7 (23%) vs. n = 10 (31%) vs. n = 6 (19%)—ongoing pregnancy rate per cycle with transfer n = 6 (16%) vs. n = 7 (18%) vs. n = 9 (23%) vs. n = 6 (15%)—live birth rate per started cycle n = 6 (23%) vs. n = 7 (23%) vs. n = 9 (28%) vs. n = 6 (19%)—live birth rate per cycle with transfer n = 6 (16%) vs. n = 7 (18%) vs. n = 9 (23%) vs. n = 6 (15%)—live birth rate at 4 weeks per started cycle n = 6 (23%) vs. n = 7 (23%) vs. n = 9 (28%) vs. n = 6 (19%)—live birth rate at 4 weeks per started cycle	[20]
n = 403	n = 297; follitropin alfa or beta n = 106; follitropin delta	8.42 (SD 4.64) vs. 7.41 (SD 3.43); mean no. of follicles ≥ 15 mm 3.94 (SD 2.14) vs. 3.60 (SD 1.87); mean no. of follicles ≥ 18 mm 1.47 (SD 1.23) vs. 1.41 (SD 1.13); mean no. of follicles ≥ 20 mm 12.3 (SD 7.7) vs. 10.4 (SD 6.1); mean no. of oocytes retrieved 0.760 (SD 0.207) vs. 0.732 (SD 0.240); proportion of normal fertilization	1951 IU (SD 849) 132 µg (SD 245)	35.3% vs. 38.7%—clinical pregnancy rate per fresh transfer cycles D3 37.6% vs. 38.5%—clinical pregnancy rate per fresh transfer cycles D5	[21]
n = 153	n = 78; follitropin delta n = 75; follitropin alfa	follicles ≥ 12 mm 2.5 ± 2.9 vs. 4.0 ± 4.0; stimulation D6 12.1 ± 7.0 vs. 18.3 ± 7.0; end of stimulation follicles ≥ 17 mm 5.2 ± 3.6 vs. 7.7 ± 4.9; end of stimulation 9.3 ± 6.7 vs. 17.9 ± 8.7; oocytes retrieved per started cycle 10.3 ± 6.2 vs. 17.9 ± 8.7; oocytes for subjects with oocyte retrieval 3.2 ± 2.9 vs. 5.9 ± 5.1; blastocysts D5	12 µg—no limit min dose 150 IU (11 µg); 75 IU adjustments	29.5% vs. 25.3%—vital pregnancy rate 28.2% vs. 24.0%—ongoing pregnancy rate	[22]
n = 347	n = 170; follitropin delta n = 177; follitropin beta	9.3 ± 5.4 vs. 10.5 ± 6.1; oocytes retrieved 7.2 ± 3.7 vs. 7.0 ± 3.4; oocytes retrieved when AMH < 15 pmol/L 10.8 ± 5.9 vs. 12.9 ± 6.4; oocytes retrieved when AMH ≥ 15 pmol/L 3.1 ± 2.7 vs. 4.2 ± 3.4; blastocysts D5	6 µg—min dose-12 µg—max dose 150 IU (15 µg); 75 IU adjustments, up to max 375 IU	n = 43 (25.3%) vs. n = 42 (23.7%)—clinical pregnancy rate per started cycle n = 43 (31.9%) vs. n = 42 (29.8%)—clinical pregnancy rate per cycle with transfer n = 40 (23.5%) vs. n = 34 (19.2%)—ongoing pregnancy rate per started cycle n = 40 (29.6%) vs. n = 34 (24.1%)—ongoing pregnancy rate per cycle with transfer n = 40 (23.5%) vs. n = 33 (18.6%)—live birth rate per started cycle n = 40 (29.6%) vs. n = 33 (23.4%)—live birth rate per cycle with transfer	[23]
n = 1009	n = 499; follitropin delta n = 510; follitropin alfa	10.0 ± 6.1 vs. 12.4 ± 7.3; oocytes retrieved in all patients 64 ± 23 vs. 64 ± 21; fertilization rate in all patients 7.0 ± 4.6 vs. 8.7 ± 5.5; embryos D3 in all patients 9.6 ± 5.3 vs. 7.6 ± 3.5; oocytes retrieved when AMH < 15 pmol/L 66 ± 22 vs. 67 ± 22; fertilization rate when FAMH < 15 pmol/L 6.8 ± 3.7 vs. 5.6 ± 2.9; embryos D3 when AMH < 15 pmol/L 10.1 ± 6.3 vs. 13.8 ± 7.5; oocytes retrieved when AMH ≥ 15 pmol/L 63 ± 23 vs. 63 ± 21; fertilization rate when AMH ≥ 15 pmol/L 7.0 ± 4.8 vs. 9.6 ± 5.7; embryos D3 when AMH ≥ 15 pmol/L	6 µg—min dose-12 µg—max dose 150 IU (11 µg); 75 IU adjustments, up to max 450 IU	n = 180 (36.1%) vs. n = 159 (31.2%)—clinical pregnancy rate n = 156 (31.3%) vs. n = 131 (25.7%)—ongoing pregnancy rate n = 156 (31.3%) vs. n = 126 (24.7%)—live birth rate n = 156 (31.3%) vs. n = 126 (24.7%)—live birth rate at 4 weeks	[24]
n = 360	-	11.2 (±6.7); no. of oocytes 69.1 (±25); rate of fertilization in two-pronuclear/metaphase II 55.5 (±24.5); rate of fertilization in two-pronuclear/oocyte 81.7 (±18.1); rate of fertilization in metaphase II/oocyte	8.7 µg—median 9.0 µg (±2.3 SD)—mean	n = 109 (38.2%)—clinical pregnancy rate in the first fresh cycle with an ET 36.8%—clinical pregnancy rate in the fresh transfer cycle with a SET 49.4%—cumulative clinical pregnancy rate for the first stimulation cycle including cryopreservation cycles 9.3%—spontaneous miscarriage rate per transfer for the first fresh cycle 13.2%—spontaneous miscarriage rate per transfer for the first stimulation cycle including cryopreservation cycles 20.7%—miscarriage rate per clinical pregnancy in the first fresh cycle 23.4%—miscarriage rate per clinical pregnancy in the first stimulation cycle including cryopreservation cycles	[25]
n = 619	n = 104; placebo n = 104; follitropin delta n = 101; follitropin delta n = 99; follitropin delta n = 107; follitropin delta n = 104; follitropin delta	12.7 vs. 11.8 vs. 11.6 vs. 11.0 vs. 11.4 vs. 10.6; follicles ≥ 12 mm 5.2 vs. 5.3 vs. 5.1 vs. 5.3 vs. 5.3 vs. 4.9; follicles ≥ 17 mm 12.5 vs. 10.6 vs. 10.7 vs. 10.6 vs. 11.3 vs. 9.7; oocytes retrieved 9.7 vs. 8.2 vs. 8.3 vs. 8.0 vs. 8.4 vs. 7.3; oocytes metaphase II 7.4 vs. 6.0 vs. 6.1 vs. 5.5 vs. 5.9 vs. 5.1; oocytes fertilized 7.4 vs. 5.9 vs. 6.1 vs. 5.5 vs. 5.9 vs. 5.1; embryos D3 5.3 vs. 4.0 vs. 4.6 vs. 3.6 vs. 4.2 vs. 3.5; blastocysts D5 3.3 vs. 2.2 vs. 3.0 vs. 2.2 vs. 2.6 vs. 2.1; good quality blastocysts D5	1 µg 2 µg 4 µg 8 µg 12 µg	42.9% vs. 28.4% vs. 30.1% vs. 41.3% vs. 40.3% vs. 35.3%—vital pregnancy rate 42.9% vs. 28.4% vs. 29.1% vs. 39.2% vs. 37.4% vs. 30.4%—ongoing pregnancy rate	[26]

SD—standard deviation, ET—embryo transfer, SET—single embryo transfer.

**Table 2:** Undergoing multinational and national projects with their settings.

Country	Number of Participants	Type of Study	Stage	Drug	Dose Concentration	Estimation Completion	Identifier	Reference
Spain n = 1 center	n = 300	Interventional (Randomized)	Phase 3	follitropin delta follitropin alfa	5 µg—min dose; 20 µg—max dose; 5 µg adjustments 225 IU; 75 IU adjustments; 75 IU—min; 300 IU—max;	2023	NCT05263388	[27]
Germany n = 1 center	n = 300	Observational (Cohort)	-	follitropin delta	NS	2024	NCT05173597	[28]
Denmark n = 1 center	n = 200	Observational (Cohort)	-	follitropin delta	NS	2024	NCT05499052	[29]
India n = 11 centers	n = 220	Interventional (Randomized)	Phase 3	follitropin delta follitropin alfa	12 µg—max 150 IU; 75 IU adjustments, up to 450 IU	2024	NCT04773353	[30]

NS—not specified.

## Data Availability

The datasets used and analyzed during the current study are available from the corresponding author on reasonable request.
